# Third-Degree Heart Block Associated With Saddle Pulmonary Embolism: A Rare Sequelae of COVID-19-Induced Hypercoagulable State

**DOI:** 10.7759/cureus.16246

**Published:** 2021-07-07

**Authors:** Zeeshan Ismail, Joshua K Salabei, Greg Stanger, Zekarias T Asnake, Leora Frimer, Andrew Smock

**Affiliations:** 1 Internal Medicine, University of Central Florida School of Medicine/Hospital Corporation of America (HCA) Graduate Medical Education Consortium/North Florida Regional Medical Center, Gainesville, USA

**Keywords:** third degree atrioventricular block, pulmonary emboli, covid 19, hypercoagulable state, saddle embolus

## Abstract

The pathophysiology of coronavirus disease 2019 (COVID-19) involves multi-organ dysfunction, particularly involving the respiratory, cardiovascular and hematological systems. This dysfunction is partly due to systemic inflammation causing a wide array of pathological sequelae thus posing a significant challenge to management despite the advances in treatment made thus far. In this report, we present a COVID-19 patient who developed a transient complete heart block and was temporarily paced as a complication of a saddle pulmonary embolus (PE). The mechanism of complete heart block is unclear, may be related to strain, ischemia, or vagal response. We believe that this is a unique sequence of events in a COVID-19 patient and, to our knowledge, is the first of its kind to be reported.

## Introduction

Coronavirus disease 2019 (COVID-19) is a unique disease that can lead to a hypercoagulable state which can cause complications of coagulation such as arterial and venous thromboembolism [1]. The proposed mechanisms of coagulopathy in COVID-19 include cytokine storm, complement activation, viral-induced coagulation activation, and endothelial injury/endothelitis [1-4]. Injury plays a central role and can occur due to direct invasion of endothelial cells by the severe acute respiratory syndrome coronavirus 2 (SARS-CoV-2) leading to cellular injury and release of inflammatory cytokines such as interleukin-6 (IL-6). The coagulopathy that ensues leads to widespread arterial/venous thrombosis; therefore, reports of pulmonary embolism (PE) in COVID-19 patients are not uncommon [1-8]. Also, cardiovascular complications are recognizable features of COVID-19 and include ischemic cardiomyopathy and arrhythmias. Proposed mechanisms underlying arrhythmias seen in COVID-19 include COVID-19-induced coagulopathy/ischemic cardiomyopathy, direct myocardial damage due to viral entry via angiotensin-converting enzyme 2 (ACE2) receptors, and vasculitis [9].

In this case, we present a 52-year-old male with COVID-19 pneumonia who developed a saddle embolus leading to significant right ventricular dilation and third-degree heart block requiring a temporary pacemaker. He was treated with heparin leading to the resolution of the heart block. We thus propose that his heart block was associated with the significant ventricular strain and deformity that improved after anticoagulation treatment. This case highlights, to the best of our knowledge, a sequelae of events initiated because of a COVID-19 hypercoagulable state leading to the development of a PE with subsequent third-degree heart block. The complexity of managing COVID-19 patients, who typically present with multi-organ dysfunction, has also been highlighted.

## Case presentation

**Figure 1:**
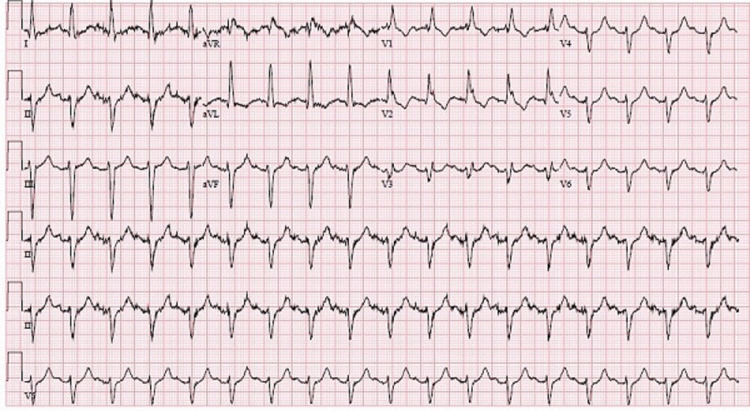
Normal sinus rhythm with bi-fascicular bundle branch block Right bundle branch block + left anterior fascicular block. Patient presented in this rhythm and returned to this rhythm after episodes of complete heart block.

**Figure 2:**
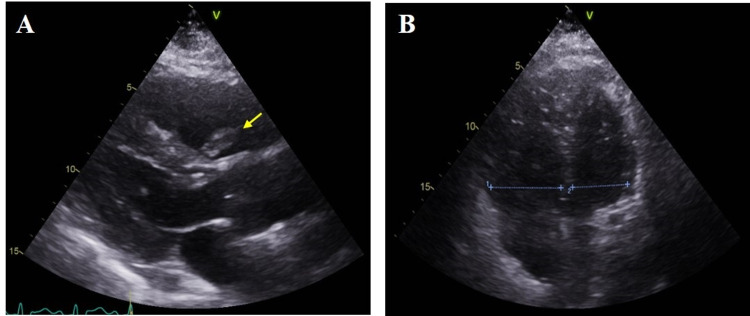
Representative ultrasound images (A) Right ventricular (RV) embolus entangled within the tricuspid apparatus (yellow arrow). Also showing minimal compression of left ventricle (LV) due to elevated RV pressures. (B) Apical four-chamber view showing an enlarged/dilated right ventricle, representing right heart strain.

**Figure 3:**
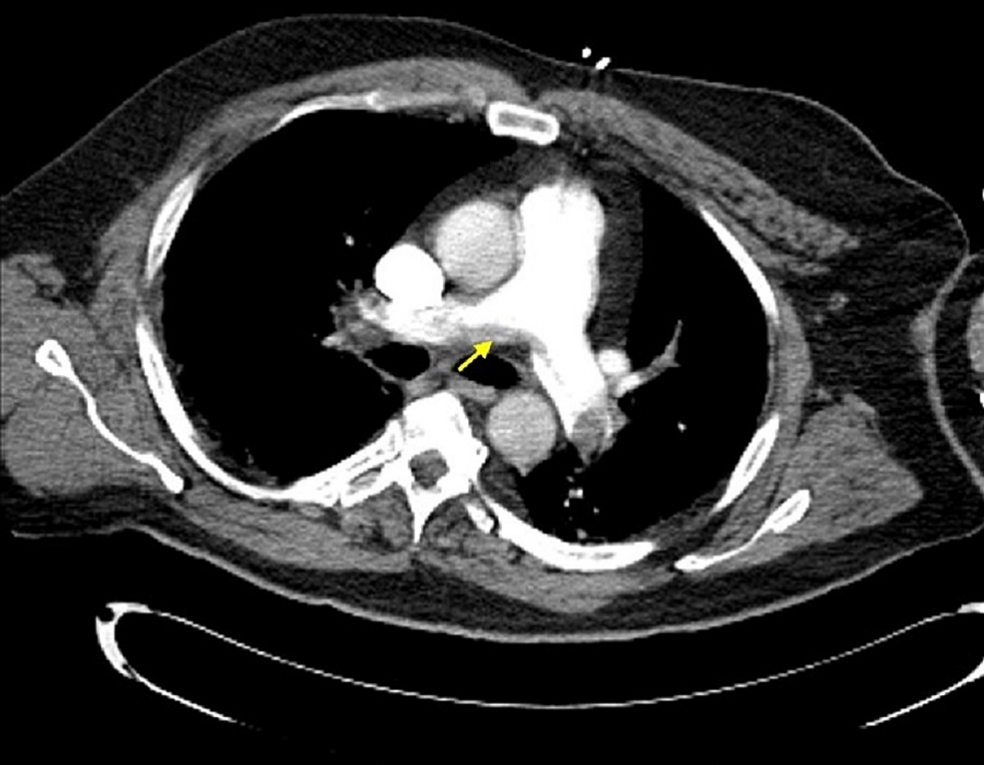
Computed tomography angiography of the chest Representative cross-sectional view. Saddle pulmonary embolus indicated by yellow arrow.

A 52-year-old male with no previous past medical history was transferred from an outside facility after presenting with complaints of three weeks of dyspnea, dry cough, poor appetite, and left-sided weakness. There, he was found to be positive for COVID-19 and evaluation, including EKG, chest computed tomography angiography (CTA), echocardiography, and ultrasound of his lower extremity showed a bifascicular block (i.e., a right bundle branch and a left anterior fascicular block), a large right ventricular (RV) embolus with associated RV dilatation, a saddle PE, and thrombi in the left popliteal and peroneal veins, respectively (Figures 1-3).

**Figure 4:**
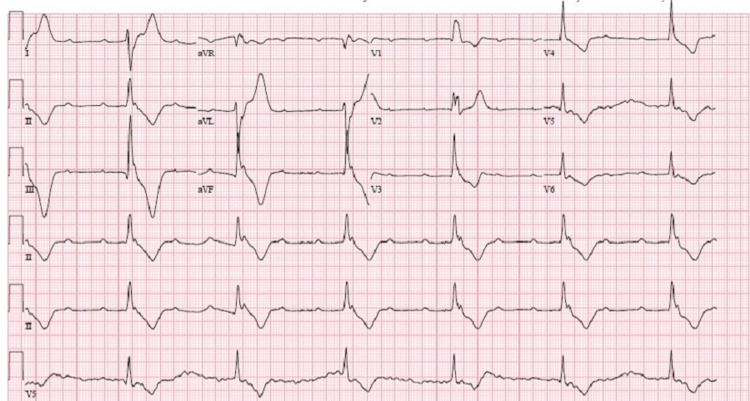
EKG showing third-degree block Notable wide QRS complexes with P and R waves marching independently.

In addition, a right middle cerebral artery (MCA) infarct was noted on brain CTA. He was started on a heparin drip and TPA was not administered because he remained hemodynamically stable and he was outside of the window for MCA reperfusion therapy. He subsequently developed a complete heart block which led to an episode of ventricular tachycardia with a palpable pulse that returned to normal sinus rhythm with amiodarone treatment (Figure 4). However, normal sinus rhythm did not persist and he returned to third-degree atrioventricular (AV) block with an idioventricular rhythm; due to persistence of this rhythm, it was decided to install a temporary right femoral trans-venous cardiac pacing device.

He was then transferred to our facility for further evaluation for possible surgical intervention. On presentation, he was oriented, however, his long-term recall and processing was impaired. His speech was dysarthric. He was afebrile, normotensive, and was saturating 100% on a non-rebreather mask; however, his heart rate was 110 beats per minute and regular. Physical exam was significant for left lower extremity hemiparesis with an otherwise grossly intact cranial nerve examination. Pertinent laboratory values obtained on presentation are shown in Table 1.

**Table 1:** Pertinent lab data on presentation to the second hospital Other lab findings included a normal metabolic panel and urinalysis. Arterial blood gas (ABG): 7.44/31/193/21.1 on 15L of oxygen via non-rebreather.

Table 1: Pertinent lab data on presentation to second hospital

Parameters	Level on admission	Normal range
White blood cell account	12.5	(4.5 - 11.0 thousand/mm^3^)
Hemoglobin	12.4	(12.0 - 15.0 g/dL)
Hematocrit	36.9	(35.0-49.0%)
Platelet count	197	150-450 thousand/mm^3^
Prothrombin time	11.7	9-12.5sec
International normalized ratio	1.1	<1.1
Partial thromboplastin time	25	21-35sec
Fibrinogen activity	25	2-19mm
Magnesium	2.4	1.7-2.2mmol/L
Lactic acid	1.6	<2
D-dimer	10.91	<0.5mg/L
Lactate dehydrogenase	649	87-241U/L
Ferritin	2082.9	20-250ng/mL
C-Reactive peptide	5.53	<5mg/L
Pro BNP	5800	0-900
Thyroid stimulating hormone	1.38	0.5-5.0 mIU/L
Vitamin D	11	30-50ng/mL

He was continued on heparin drip and other therapies to treat his COVID-19. Repeat imaging studies did not show any significant change from findings seen at the outside facility. Cardiothoracic surgery was then consulted for surgical evaluation; however, they recommended against any surgical intervention. Ultimately, a permanent pacemaker was not indicated as the patient remained in sinus rhythm throughout the remainder of his hospitalization. He was then discharged to a subacute care facility on long-term anticoagulation with warfarin.

## Discussion

Herein, we have presented a case of complications resulting from COVID-19-induced hypercoagulable state. Our patient presented with a heavy clot burden as evidenced by clots in his lower extremity veins, right ventricle, lungs, and MCA. This case highlights the possible widespread arterial and venous thrombosis seen in COVID-19 patients.

The common EKG presentations of acute PE usually include sinus tachycardia, S1Q3T3, negative T wave in lead 3, incomplete or complete right bundle branch block [10-11]. EKG findings generally reflect reactive compensation and right heart strain. The development of complete heart block in a hemodynamically stable patient with a large PE is rare and has only been reported in a few case reports [12-13].

The right bundle branch may be implicated due to its superficial pathway in the right ventricle and can be compromised in the setting of right ventricular strain due to increased afterload caused by a PE. The lower pressure pulmonary circulation is not accustomed to higher pressures and is more sensitive to afterload increases, especially in the acute obstructive setting of a PE. However, the direct mechanism leading to complete AV block in our patient is unclear partly because we did not have any prior EKGs for comparison. However, the patient had no reported past medical history and he specifically denied prior chest pain or hospitalizations secondary to acute coronary syndrome. Therefore, it is highly unlikely that he had previous cardiac conduction system pathology that was exacerbated by the presence of a large PE. Thus, one mechanism leading to the current complete heart block is that the massive PE significantly obstructed blood flow such that it limited coronary artery filling and subsequently AV nodal ischemia. Another possible explanation is the proposed mechanism of the Bezold-Jarisch reflex leading to transient bradycardia or complete AV block [11-13]. Also possible, would be the severe right heart strain and dilation directly compromising Purkinje fibers and other cardiac conduction apparatus. Interestingly, we could not find any prior observational or experimental studies on the development of complete heart block from a PE.

## Conclusions

Herein, we have presented a unique case of a COVID-19 patient who developed lower extremity deep vein thrombosis leading to clot migration to the right ventricle and pulmonary artery. Interestingly, this clot burden was significant enough to cause a saddle embolus and a presenting EKG involving a right bundle branch and left anterior fascicular block. The transient involvement of the left posterior fascicle leading to a complete heart block is a rare finding in this setting and incompletely understood. Further, this is a unique scenario where a permanent pacemaker may not be necessary for a third degree AV block, as the arrhythmia is expected to resolve with resolution of the embolus. This is the first case, to our knowledge, reporting this series of events in a COVID-19 patient.
